# Role of Extracellular Vesicles in Autoimmune Pathogenesis

**DOI:** 10.3389/fimmu.2020.579043

**Published:** 2020-09-23

**Authors:** Wen-Cheng Wu, Sheng-Jiao Song, Yuan Zhang, Xing Li

**Affiliations:** National Engineering Laboratory for Resource Development of Endangered Crude Drugs in Northwest China, The Key Laboratory of Medicinal Resources and Natural Pharmaceutical Chemistry, The Ministry of Education, College of Life Sciences, Shaanxi Normal University, Xi'an, China

**Keywords:** autoimmune disease, extracellular vesicle, cell communication, biomarker, pathogenesis

## Abstract

Autoimmune diseases are conditions that emerge from abnormal immune responses to natural parts of the body. Extracellular vesicles (EVs) are membranous structures found in almost all types of cells. Because EVs often transport “cargo” between cells, their ability to crosstalk may be an important communication pathway within the body. The pathophysiological role of EVs is increasingly recognized in autoimmune diseases, including multiple sclerosis, rheumatoid arthritis, systemic lupus erythematosus, Sjogren's syndrome, Type 1 diabetes, and autoimmune thyroid disease. EVs are considered as biomarkers of these diseases. This article outlines existing knowledge on the biogenesis of EVs, their role as messegers in cellular communication and the function in T/B cell differentiation and maturation, and focusing on their potential application in autoimmune diseases.

## Introduction

**Figure 1:**
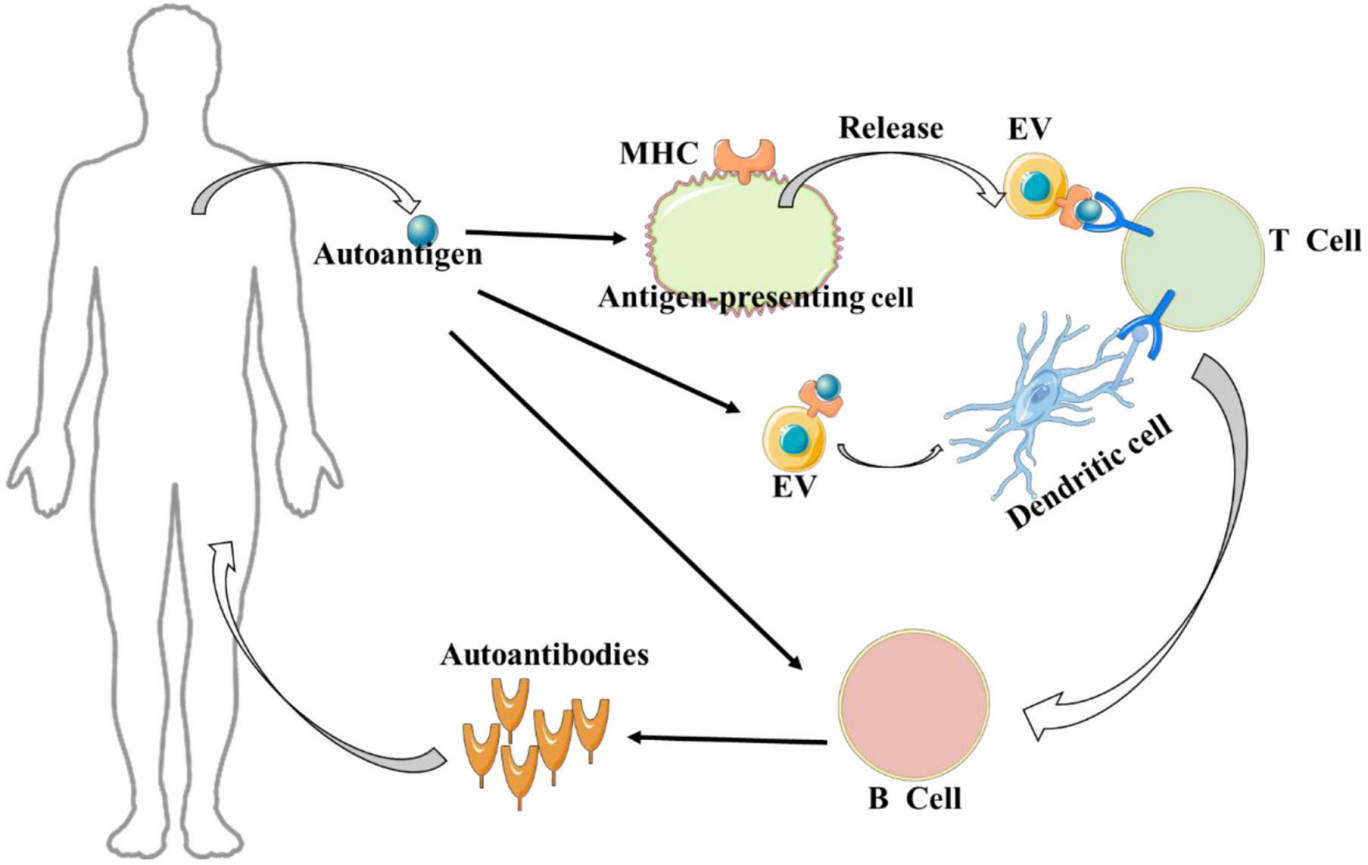
Extracellular vesicles (EVs) and autoimmune disease. It is worth noting, on the one hand, autoantigens can be captured by antigen-presenting cells and transmitted to extracellular vesicles to activate T cells; on the other hand, autoantigens can be directly transmitted to extracellular vesicles to activate T cells in the presence of dendritic cells.

Autoimmune diseases are the result of interactions between genetic and environmental factors that cause an immune response to self-produced antigens in the body. This then leads to self-damage of tissues or organs. In normally functioning immune systems, there are various tolerance mechanisms that play a protective role in preventing an autoreactive lymphocyte response (1). In autoimmune diseases, immune cell tolerance mechanisms become problematic, leading to the stimulation of autoreactive T and B lymphocytes (Figure 1) (2–5). Additionally, the interaction of various inflammatory cytokines and chemokines can lead to an imbalance between regulatory (e.g., Tregs) and inflammatory cells (e.g., Th17 cells), as well as abnormal autoantigen clearance mechanisms and antigen presentation, all of which can result in the development of autoimmune diseases (6, 7).

## Extracellular Vesicles

### Definition and Classification

Extracellular vesicles (EVs) are a collective term for phospholipid bilayer structures secreted by cells, which typically contain proteins, mRNA, miRNA, and/or other substances. EVs are normally present in body fluids, and can be released by nearly all cell types (8, 9). EVs are categorized according to their morphology, size, and biogenesis. EVs with a diameter of 50–2000 nm and produced by the process of apoptosis are called apoptotic bodies (10); EVs with a diameter of approximately 100–1000 nm and produced by budding or fission of the plasma membrane are called microvesicles; and EVs with a diameter of 50–150 nm, which are produced by exocytosis of multivesicular endosomes (MVEs) are called exosomes (11). MVE biogenesis may involve several parallel pathways, specifically, the endosomal sorting complex required for transport (ESCRT) dependent process requiring sphingolipids, and the ESCRT-independent manner, involving tetraspanins (12). EV morphology is also very diverse, with various exosomes observed within and on the surface of cells. These exosomes include various proteins, such as TSG101, ALIX, Integrin-1, CD9, CD63, CD81, and CD82 (13, 14).

### Biological Functions

Although almost all cell types generate EVs and deliver them into the extracellular space, the biological activities of EVs derived from different cells can vary greatly. Specifically, exosomes can be involved in various disorders, such as cancer, neurodegeneration, and inflammatory disease (15, 16). In the central nervous system (CNS), EVs participate in the communication process among neurons, glia, and microglia (8). In certain pathological situations, EVs are viewed as mediators of disease. Microglia-derived EVs have been found to be actively involved in neuroinflammation and neurodegenerative diseases (17). There is evidence that EVs play a role in cell-cell communication by carrying multiple pieces of information in the form of membrane proteins, carbohydrates, and lipids, as well as other molecules that require protection from extracellular enzyme degradation, such as RNA, proteins, and metabolites (18). EVs also facilitate communication between infected cells and interactions between host cells and bacteria. Accumulating evidence suggests that EVs bearing damage-associated molecular patterns (DAMPs) secreted from stressed or injured tissues play an important role in inflammation (19, 20). Recent studies have shown that EVs possess the ability to stimulate immune responses, play a critical role in the pathogenesis of autoimmune diseases, and have great potential as biomarkers for the detection of autoimmune diseases and as therapeutic agents. For example, IFN-α and TNF-α encapsulated in EVs from patients with systemic lupus erythematosus (SLE) are significantly higher than in healthy individuals, which means that detection IFN-α and TNF-α levels in EVs of patients can be used as novel diagnostic markers of SLE (21). Long non-coding RNA (lncRNA) in EVs can take the lead in immune cell development and activation through interactions with the nucleotide or proteins. However, lncRNA dysregulation is found in many autoimmune diseases (22). Indeed, EVs may be involved in the presentation of intracellular self-antigens to the immune system. Antigen-presenting cells (APCs) can undergo intracellular transfer and effective antigen delivery to autoantigen-specific T cells through EVs, which represents a highly efficient mechanism of antigen presentation (23–25). Additionally, many types of tissue- or humoral-derived EVs have been discovered to possess immunomodulatory or tolerogenic activities. For instance, Robins et al. found that EVs derived from plasma or serum *in vivo* can alleviate chronic inflammation development (6). This suggests that EVs have the potential to treat autoimmune diseases.

## EVs and Cellular Communication

The crosstalk between cells through EVs may be an important communication pathway, particularly in autoimmune diseases. In rheumatoid arthritis (RA), synovial fibroblast-derived EVs can induce inflammatory changes in chondrocytes (26). Conversely, in synovial fluid cells (including monocytes and granulocytes), EVs can regulate synovial fibroblast cytokine secretion by increasing the release of monocyte chemokines and cytokines, which, in turn, leads to worsening inflammation (27). EVs play an active role in the pathogenesis of multiple sclerosis (MS). Specifically, it has been shown that EVs released from blood-brain barrier (BBB) endothelial cells are able to stimulate CD4^+^ and CD8^+^ T cell activation with no stimulatory signal, which indicates that EVs may enhance T cell activation and subsequent antigen presentation (28). Moreover, EVs also play a key role in communication between immune cells. Despite the fact that EVs can regulate the biological activity of many types of immune cells, including T cells and natural killer cells (NK cells), the most effective regulatory activity of EVs is granted by APCs (6). APCs primarily control immune function through membrane proteins—specifically, MHC class I and II molecules; costimulatory molecules, such as CD80, CD86; and adhesion molecules (29–31). Similarly, EVs derived from APCs are involved in this process (25). In contrast, endogenous EVs are the source of autoantigens and can activate autoreactive T cells. Further, they are involved in the formation of immune complexes to stimulate autoimmunity and therefore mediate autoimmune diseases (6, 32). It has been reported that miRNAs can be functionally delivered to recipient cells, so the role of intracellular miRNAs is also considered an important modulator of gene expression in immune cells (33). In EVs, miRNAs have also been shown to possess immune functions. It has been demonstrated that EVs will fuse with target dendritic cells (DCs) and subsequently release their contents into the DC cytosol. Furthermore, transfer of miRNAs by EVs were shown to inhibit target mRNAs of recipient DCs. These findings suggest a role for EVs in communication and post-transcriptional regulation between DCs (34).

## Extracellular Vesicles Involved in the Differentiation and Maturation of T/B Cells

As mentioned earlier, EVs are closely related to the immune system. In the immune system, NK cells are important to the function of innate immunity, whereas B cells, as well as T cells, are essential part of adaptive immunity. B cells distinguish foreign antigens, but T cells require APCs to recognize antigens. After antigen recognition, MHC-I and MHC-II present it to CD8^+^ and CD4^+^ T cells, thereby activating the immune response (35). Many researches have demonstrated that immune cells release immunocompetent EVs, which exert a critical function in innate and adaptive immune process, including antigen presentation, NK/T cell activation, T cell polarization, and immunosuppression (36). EVs released by APCs or B cells express MHC-I, MHC-II, and T cell costimulatory molecules that act as antigen presentation platforms to activate CD8^+^ and CD4^+^ T cells (25, 37). In addition, antigens transported by EVs can form complexes with MHC through APCs, and antigen-carrying EVs can directly promote T cell activation in the presence of naive DCs (38). There is evidence that macrophages with neutrophil-derived EVs have the capacity to inhibit Th1 and Th2 cell differentiation, as well as the ability to induce Treg cells. In addition, under certain circumstances, it can also promote Th17 cell polarization, restrict CD8^+^ T cell and B cell function, and affect DC migration, resulting in effectively attenuating T cell responses (39). In fact, EVs derived from innate immune cells mostly mediate the development of effector T cells by targeting DCs and subsequent delivery of antigen or affecting DC activation/migration (40). Immune cell-derived EVs not only promote immunity but can also reduce immune activity. For example, EVs released by T cells can target many other types of cells and induce various immune reactions that range from immune activation to inhibition (41).

## EVs and Autoimmune Diseases

### Multiple Sclerosis

Multiple sclerosis (MS) is a chronic autoimmune disease affecting the CNS and characterized by inflammatory demyelination. Its pathogenesis primarily involves genetic, environmental, and immune components (42). MS is an inflammatory cascade triggered by autoreactive effector T cells and is associated with immune cells such as B cells, macrophages, and natural killer cells (43–46). It has been found that medullary EVs present in the cerebrospinal fluid are derived from microglia, and microglia will generate and release IL1-β and MHC-II, indicating that EVs secreted by reactive myeloid cells may trigger neuroinflammation and contribute to the rapid spread and presentation of antigens (47). Conversely, EVs can cross the BBB to affect the migration of immune cells, which, in turn, promotes disease progression in MS. For example, microglia, astrocytes, and platelets shed exosomes containing metalloproteinases and caspase-1 in response to stimulation by proinflammatory cytokines, such as TNF. It is well-known that these enzymes are able to induce BBB disruption and promote lymphocyte and myeloid cell migration into the CNS (48, 49). Meanwhile, in MS patients, Barry et al., found that EVs released from activated T cells can promote monocyte recruitment and upregulate intercellular cell adhesion molecule-1 (ICAM-1) in endothelial cells as well as macrophage-1 antigen (Mac-1) expression in monocytes. Activated monocyte expression of Mac-1 integrin and its binding to ICAM-1 is an essential step in the transendothelial migration of inflammatory cells (50). Brain endothelium-derived microvesicles have been shown to be involved in the activation of CD4^+^ and CD8^+^ T lymphocytes through the expression of MHCII and CD40 molecules (51), suggesting that EVs are involved in the pathogenesis of MS. Azimi et al., found that miR-326a to be overexpressed in T cell-derived exosomes from MS patients (52). Furthermore, it is well-known that miR-326 is upregulated in MS and affects the CD47 molecule, which, in turn, suppresses the activity of macrophages and decreases their expression, thereby increasing the phagocytosis of myelin (48). Further, miR-326 can promote naïve T cell differentiation into Th17 cells by targeting negative regulators of Th17 polarization, thus increasing the severity of MS (53).

### RA

RA is a chronic inflammatory autoimmune disease characterized by swelling, tenderness, and destruction of synovial joints. It has been shown that EVs are associated with immune complex formation, antigen presentation, miRNA delivery, activation of fibroblast-like synoviocytes (FLS), intercellular communication, and degradation of the extracellular matrix in the pathogenesis of RA (54). EVs derived from FLS have been found to load citrullinated proteins in their membranes, such as macrophage apoptosis inhibitory factor (AIM), which can stimulate the formation of immune complexes (55, 56). These EVs contain antigens, antibodies, and complement immune complexes, it has been shown that complement may exert an important function in the pathogenesis of RA. Synovial fluid (SF)-derived EVs can activate complement and, in fact, many proinflammatory products are released once the complement cascade is activated, thereby inducing joint inflammation in rheumatic diseases (57). EVs encapsulating DNA-binding proteins-DEK are known to present this antigen to CD8^+^ T cells and NK cells. This can lead to more efficient antigen presentation and enhanced immune system activation (55). TNF-α is contained in FLS-derived EVs isolated from RA patients, and results in NF-κB activation, which can promote inflammation (58). In addition, monocyte or T-cell derived EVs enhance the release of matrix metalloproteinases (MMPs; MMP-1, MMP-3, MMP-9, and MMP-13) from FLS through an NF-κB-dependent pathway. MMPs break down proteoglycans in the extracellular matrix and is a principal mechanism of cartilage destruction in RA (59, 60). It is well-known that miRNAs in EVs play an important role in mediating cell-to-cell communication. MiR-155 and miR-146a have been the most studied in the current pathology of RA. Studies have demonstrated that miR-155 and miR-146a in DC-derived exosomes can be taken up by immune cells and then affect intercellular communication, in which miR-155 can upregulate the expression of TNF-α and IL-6 inflammatory genes, whereas miR-146a inhibits the expression of TNF-α and IL-6 inflammatory genes. Further studies have indicated that exosome-derived miR-155 and miR-146a secreted by immune cells can mediate information and substance exchange between FLS and other immune cells, thereby regulating the disease process of RA (61). In addition, some studies have shown that RA patients are positively correlated with the prevalence of cardiovascular disease. At present, the well-known pathogenesis hypothesis of atherosclerosis in RA is the chronic inflammation which proposed by Ross (62). Since their discovery, several studies have been evidenced micropaticles (MPs) exert important function in atherosclerosis including endothelial derived MPs (EMPs), and Tang cells derived MPs (Tang-MPs) (62). EMPs released after endothelial cell dysfunction can make the endothelial permanent damage, leading to the activation of various pathways, such as matrix metalloproteinase-2 (MMP-2) (63) and inducible NO synthase (iNOS) activation (64); expression of E-selectin, ICAM-1, and VCAM-1; and reactive oxygen spices (ROS) formation (65). Moreover, the newly discovered found an association between TNF-α and Tang MPs levels (66). TNF-α is the main cytokine involved in the pathogenesis of RA, which can lead to atherosclerosis and endothelial dysfunction (67, 68). It has been demonstrated that MPs in RA patients express TNF-α on their surface (69), Barbati et al., showed that MPs can induce the activation of asparaginase 3 and TNF-related apoptosis-inducing ligand (TRAIL) and TNF receptor, three independent signals that promote endothelial cell apoptosis, which can lead to the formation of atherosclerosis (62, 70). All the above literatures indicate that extracellular vesicles in RA are not only involved in its pathogenesis, but also mainly related to inflammation, atherosclerosis, and impaired endothelial function.

### SLE

SLE is a chronic autoimmune disease that is clinically heterogeneous and affects different organs. Like many autoimmune diseases, SLE is associated with genetic factors, and environmental factors. The main mechanism is due to the production of antibodies against self-antigens, which form the deposition of immune complexes (71, 72). Epigenetic dysregulation, such as DNA methylation (73) and histone acetylation (74), is found in many SLE patients, and is therefore considered crucial in the development of the disease. Additionally, environmental factors may also trigger autoimmune responses that can lead to disease (75). Because EVs contain self-antigens, they can participate in the formation of immune complexes. In SLE patients, higher numbers of immunoglobulin-carrying plasma-EVs were found than in healthy individuals and, interestingly, platelet-derived EVs have been found to be a major contributor to autoimmune responses in SLE (76). Furthermore, carriage of immunoglobulins in EVs is associated with autoantibodies and complement activation, and their number correlates with anti-DNA levels (77). Lee et al. showed that serum-derived exosomes isolated from SLE patients were able to provoke a high level of cytokine generation in healthy peripheral blood mononuclear cells causing a proinflammatory response (21). Others have also shown increased expression of costimulatory surface molecules and proinflammatory cytokines, such as MHC-I IL-6, TNF-α, and IFN-α in blood-derived plasmacytoid dendritic cells and myeloid dendritic cells, when apoptotic endothelial microparticles are extracted from the plasma of SLE patients (78). The increased expression of these factors suggest a role for apoptotic endothelial microparticles in the autoimmune response of SLE patients, as well as in other inflammatory diseases. The main cause of morbidity and mortality in SLE is lupus nephritis (LN) (79). In some studies, EV-associated miRNAs are proposed as biomarkers of renal damage in SLE (80). For example, miR-29c levels have been reported to be decreased in LN patients, when compared with controls. These levels correlate with renal function and the degree of renal fibrosis (81). Moreover, miR-146a has been found to be an important substance leading to changes in the type 1 interferon (IFN) pathway in SLE patients (82). Taken together, these studies reveal the possibility of miRNAs in EVs as markers of SLE and playing an important role in SLE pathogenesis.

### Sjogren's Syndrome

Sjogren's syndrome (SS) is a chronic inflammatory autoimmune disease involving exocrine glands, such as salivary and lacrimal glands, which has a high incidence in women and can be divided into two categories: primary and secondary (83). There are many antibodies against self-antigens in patients with SS, which in turn cause autoimmune reactions, leading to morbidity. Recently, EVs have been found to contain autoantigens, including Ro/SSA, La/SSB, and Sm RNPs in exosomes, and that these antigens are significantly expressed in the exosomes of SS patients (84). Studies have also demonstrated that the autoimmune response to RNP in SS is an important feature, although the exact mechanism by which these intracellular antigens enter and stimulate the immune system is unknown. However, apoptosis through the capture of apoptotic vesicles containing autoantigens by APCs to generate an autoimmune response was shown to be the main pathway (85). Kapsogeorgou et al. found that exosomes isolated from salivary glands could reflect the physiological status and regulatory level of the gland (84). Therefore, salivary gland-derived exosomes could be used as a diagnostic marker for SS disease. Michael et al., confirmed that there have significant differential expression of miRNA in salivary gland-derived exosomes from SS patients vs. healthy controls (86). These studies suggest the potential of miRNAs in the salivary glands as a diagnostic marker of SS and requires further investigation.

### Type 1 Diabetes

Type 1 diabetes (T1D) is a specific autoimmune disease of the islets of Langerhans, which is characterized by infiltration of T cells into the islets, which, in turn, leads to destruction of insulin-producing β cells (87). Activation of IFN-γ-producing Th1 cells has been found to be a key factor in the autoimmune devastation of the islets in T1D patients. In addition, it has also been shown that islet mesenchymal stem cells (MSC)-derived exosomes from non-obese diabetic (NOD) mice activate autoreactive T cells to release IFN-γ more than B6 mouse-derived exosomes (88). This suggests that MSC-derived exosomes play a key role in the autoimmune destruction of T1D disease. The paracrine action in islets can promote hormone secretion and islet survival. Recently, it has been reported that exosomes have similarly important functions in the islet (89, 90). When β-cells are exposed to proinflammatory factors, exosomes are released, which results in apoptosis of surrounding cells, and exacerbates the development of inflammation (91). There is evidence that exosomes exert a function in the initiation of autoimmune responses in pancreatic islets. For example, autoantigens (e.g., GAD65, proinsulin) are found in exosomes released from rat and human pancreatic β-cells, and are subsequently taken up by activated DCs, thereby activating autoreactive T and B cells (92). In summary, exosomes not only participate in the development of the disease but also emerge as probable diagnostic markers of T1D disease (93).

### Autoimmune Thyroid Disease

Autoimmune thyroid disease (AITD) is a common autoimmune disease with two main clinical manifestations: autoimmune thyroiditis (AT) and Graves' disease (GD) (94). In AITD, its immune-regulatory function is impaired, which leads to lymphocytic infiltration of the thyroid gland, which in turn produces antibodies against thyroid antigens, leading to thyroid dysfunction (95). An association between AITD and other organ-specific autoimmune diseases has been reported, especially in rheumatoid arthritis, systemic lupus erythematosus, systemic sclerosis, type 1 diabetes, and other diseases with a relatively high prevalence, suggesting that patients can regularly examine the thyroid gland and reduce the risk of the disease (96, 97). AITD causes immune activation which leads to partial cell activation or apoptosis. In this process, small vesicles are released from the cell membrane and are called microvesicles (MVs). MVs circulate in the bloodstream, which in turn triggers thrombosis and inflammation (98). There is evidence that the cell number of Treg and Th17 plays an important role in the pathogenesis of autoimmune diseases (99). In one study, it was found that MVs from AITD patients were able to inhibit Treg cell differentiation, and that only MVs from AITD could induce IFN-γ expression as well as differentiation of IL-17^+^IFN-γ^+^ double-positive lymphocytes (100). MiR-146a and miR-155 are important regulators of immune responses, are essential for the function and development of Treg cells, and contribute to Th17 cell function (101, 102). Only experiments have demonstrated a significant rise in miR-146a, miR-200a, and miR-155 in CD4^+^ T cells from GD patients (103). In summary, the extracellular vesicles represented by MVs have a crucial role in AITD pathogenesis, and are expected to be diagnostic markers for AITD.

**Figure 2:**
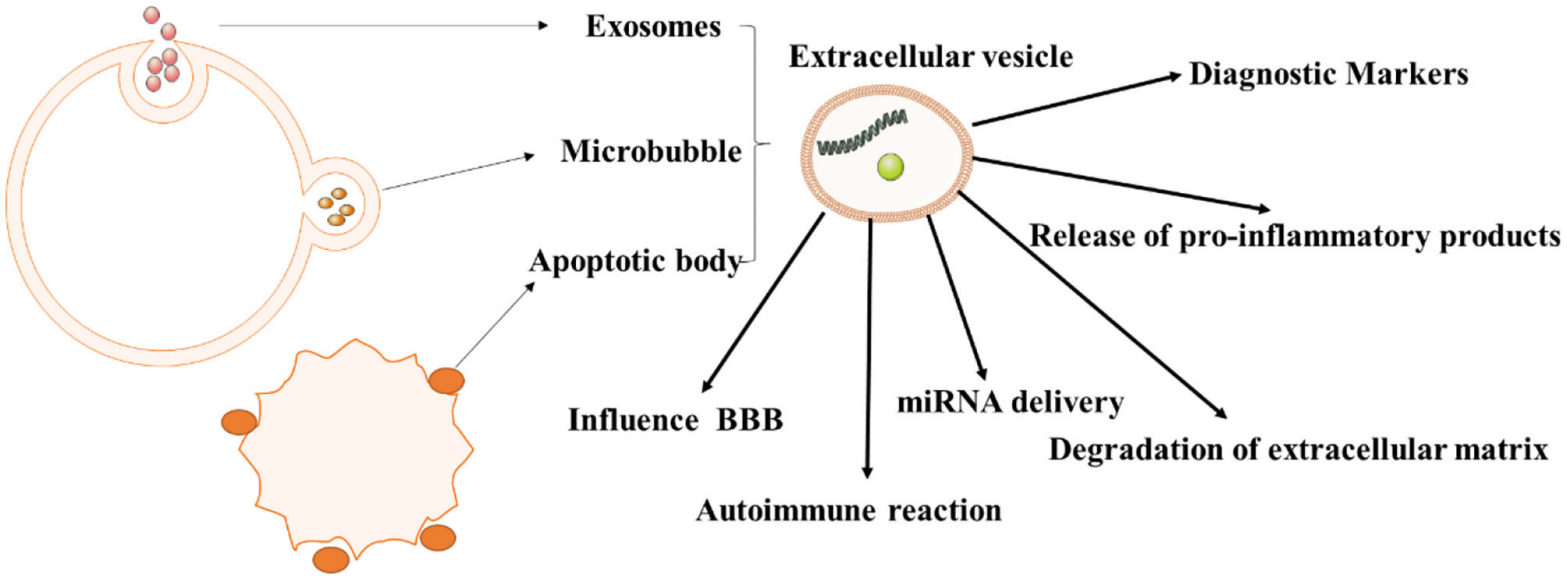
The role of extracellular vesicles in autoimmune diseases. As we all know, extracellular vesicles can be divided into exosomes, microvesicles, apoptotic bodies, and tumor vesicles. And they have different roles in the pathogenesis of autoimmune diseases, especially in immune response, inflammation.

In short, all types of EVs participate in the pathogenesis of autoimmune diseases, as shown in Figure 2. Additionally, the role of EVs in these diseases varies, as shown in Table 1.

**Table 1:** EVs involved in autoimmune diseases.

**Triggered behavior**	**Source of EV**	**MicroRNA**	**Disease**	**References**
Neuroinflammation	Microglia	None	MS	(104)
Inflammatory cell migration	T cell	None	MS	(105)
Increased myelin phagocytosis	T cell	miR-326a	MS	(53)
Stimulates formation of immune complexes	FLS	None	RA	(55, 56)
Joint inflammation	SF	None	RA	(106)
Antigen presentation	DEK	None	RA	(55)
Affects cell communication	DC	miR-155/ miR-146a	RA	(61)
Inflammatory response	Serum	None	SLE	(21)
Expression of proinflammatory factors	Plasma	None	SLE	(78)
Altered IFN pathway	None	miR-146a	SLE	(107)
Reflect physiological status	Salivary gland	None	SS	(84)
Release of IFN-γ	MSC	None	T1D	(88)
Activation of autoreactive T/B cells	Islet β cells	None	T1D	(92)
Suppression of Treg differentiation	None	miR146a/ miR-155	AITD	(100, 101)

## Conclusions and Prospects

In general, EVs are involved in the occurrence and development of the above autoimmune diseases. As EVs are released by almost all cells, they participate in many important physiological activities in the body, especially in cell-to-cell communication and activation of immune cells. Most of the causes of autoimmune diseases are due to abnormal activation of the autoimmune system, and most of the EVs involved in the pathogenesis of autoimmune diseases do so through the presentation of antigens to activate autoreactive T cells, which, in turn, mediates development of the disease. Moreover, EVs contain specific miRNAs, which are involved in the development of diseases by targeted delivery of miRNAs to the recipient cells. The important role of EVs in the process of disease development suggests to us the possibility of EVs as biomarkers. Many patients with autoimmune diseases exhibit EVs that are different from EVs released by healthy individuals. Additionally, EVs are easily isolated, stable, and contain specific molecular markers. The role EVs in autoimmune diseases should be the subject of future study, so as to enhance the prospects for the treatment and diagnosis of autoimmune diseases.

## Author Contributions

All authors listed have made a substantial, direct and intellectual contribution to the work, and approved it for publication.

## Conflict of Interest

The authors declare that the research was conducted in the absence of any commercial or financial relationships that could be construed as a potential conflict of interest.
